# Establishment of a Novel Anti-CD44 Variant 10 Monoclonal Antibody C_44_Mab-18 for Immunohistochemical Analysis against Oral Squamous Cell Carcinomas

**DOI:** 10.3390/cimb45070333

**Published:** 2023-06-21

**Authors:** Kenichiro Ishikawa, Hiroyuki Suzuki, Mika K. Kaneko, Yukinari Kato

**Affiliations:** 1 Department of Molecular Pharmacology, Tohoku University Graduate School of Medicine, 2-1 Seiryo-machi, Aoba-ku, Sendai 980-8575, Japan; ken.ishikawa.r3@dc.tohoku.ac.jp (K.I.); k.mika@med.tohoku.ac.jp (M.K.K.); 2 Department of Antibody Drug Development, Tohoku University Graduate School of Medicine, 2-1 Seiryo-machi, Aoba-ku, Sendai 980-8575, Japan

**Keywords:** CD44, CD44v10, monoclonal antibody, oral squamous cell carcinoma, immunohistochemistry

## Abstract

Head and neck squamous cell carcinoma (HNSCC) is the most common type of head and neck cancer, and has been revealed as the second-highest expression of CD44 in cancers. CD44 has been investigated as a cancer stem cell marker of HNSCC and plays a critical role in tumor malignant progression. Especially, splicing variant isoforms of CD44 (CD44v) are overexpressed in cancers and considered a promising target for cancer diagnosis and therapy. We developed monoclonal antibodies (mAbs) against CD44 by immunizing mice with CD44v3–10-overexpressed PANC-1 cells. Among the established clones, C_44_Mab-18 (IgM, kappa) reacted with CHO/CD44v3–10, but not with CHO/CD44s and parental CHO-K1 using flow cytometry. The epitope mapping using peptides that cover variant exon-encoded regions revealed that C_44_Mab-18 recognized the border sequence between variant 10 and the constant exon 16-encoded sequence. These results suggest that C_44_Mab-18 recognizes variant 10-containing CD44v, but not CD44s. Furthermore, C_44_Mab-18 could recognize the human oral squamous cell carcinoma (OSCC) cell line, HSC-3, in flow cytometry. The apparent dissociation constant (*K*_D_) of C_44_Mab-18 for CHO/CD44v3–10 and HSC-3 was 1.6 × 10^−7^ M and 1.7 × 10^−7^ M, respectively. Furthermore, C_44_Mab-18 detected CD44v3–10 but not CHO/CD44s in Western blotting, and endogenous CD44v10 in immunohistochemistry using OSCC tissues. These results indicate that C_44_Mab-18 is useful for detecting CD44v10 in flow cytometry and immunohistochemistry.

## 1. Introduction

Head and neck cancer is the seventh most common cancer type globally, and exhibits a profound impact on patients and their quality of life after surgical ablation and therapies [1]. Head and neck squamous cell carcinoma (HNSCC) is the most common type of head and neck cancer. The treatment of HNSCC includes surgery, chemotherapy, radiation therapy, immunotherapy, molecular targeted therapy, or a combination of those modalities [2]. Although survival can be improved through the development of treatments, cancer metastasis and resistance to drugs remain the main causes of death [3]. The rate of 5-year survival remains stagnant at approximately 50% [4].

CD44 is a multifunctional type I transmembrane glycoprotein that mediates metastasis and drug resistance in tumor cells. HNSCC is the second-highest CD44-expressing tumor in the Pan-Cancer Atlas [5]. The alternative splicing of CD44 mRNA produces the various isoforms [6]. The constant exons including the first five (1 to 5) and the last five (16 to 20) are present in all CD44 variants and make up the standard isoform (CD44s). The CD44 variant (CD44v) isoforms are generated by the alternative splicing of variant exons (v1 to v10) with the constant exons of CD44s [7]. Both CD44s and CD44v (pan-CD44) attach to the extracellular matrix, including hyaluronic acid (HA), and facilitate the activation of metastasis-associated intracellular signaling pathways [8].

Tumor metastasis includes multiple processes called the invasion–metastasis cascade. The processes contain (i) dissemination from primary sites, (ii) the acquisition of migration/invasion phenotype, (iii) intra/extravasation, (iv) survival in circulation, and (v) adaptation and colonization in distant organs [9]. Moreover, (vi) cancer-associated fibroblasts and tumor-infiltrating lymphocytes in the tumor microenvironment involve in the promotion of the invasion–metastasis cascade [10]. CD44 mediates the multiple steps of the invasion–metastasis cascade through interaction with HA [11] and CD44v-specific functions [12].

CD44 has been studied as a marker of cancer stem-like cells (CSCs) in tumors [13]. Anti-CD44s or CD44v monoclonal antibodies (mAbs) are used to collect the CD44-high CSCs [13]. The CD44-high population showed the increased self-renewing property, resistance to drugs, and metastatic colonization *in vivo* [13]. CD44 is the first applied CSC marker to isolate CSCs derived from HNSCC [14]. Notably, CD44-high CSCs from HNSCC showed the features of epithelial to mesenchymal transition (EMT). The EMT program activation confers tumor cells the stemness and the ability to migrate, invade, and extravasate [15]. Moreover, CD44-high cells could make colonization in the lungs of immunodeficient mice, compared to CD44-low, which failed to form the metastatic colonization [16].

Furthermore, CD44v8–10 mediates the resistance to treatment. The v8–10-encoded region binds to and stabilizes a cystine–glutamate transporter (xCT), which enhances cystine uptake and glutathione synthesis [17]. The elevation of reduced glutathione (GSH) mediates the defense to reactive oxygen species (ROS) [17], radiation [18], and chemotherapeutic drugs [19]. The expression of CD44v8–10 is associated with the xCT-mediated redox status and the poor prognosis of patients [18]. Therefore, the establishment of each CD44v-specific mAb is essential to reveal the function and develop CD44-targeting cancer therapy. However, the tissue distribution or function of the variant 10-containing CD44 has not been fully understood.

In our previous work, we developed an anti-pan-CD44 mAb, C_44_Mab-5 (IgG_1_, kappa) [20] using the Cell-Based Immunization and Screening (CBIS) method. Additionally, another anti-pan-CD44 mAb, C_44_Mab-46 [21], was established by immunizing mice with the CD44v3–10 ectodomain. Both C_44_Mab-5 and C_44_Mab-46 have the epitopes within the constant exon 2- and 5-encoding sequences [22,23,24] and could be applied to immunohistochemistry in oral squamous cell carcinomas (OSCC) [20] and esophageal SCC [21], respectively. Furthermore, we produced a class-switched and defucosylated type of recombinant C_44_Mab-5 (5-mG_2a_-f) using fucosyltransferase 8 (Fut8)-deficient ExpiCHO-S cells and investigated the antitumor activity in OSCC xenograft-transplanted mice [25]. We have developed various anti-CD44v mAbs, including C_44_Mab-6 (an anti-CD44v3 mAb) [26], C_44_Mab-108 (an anti-CD44v4 mAb) [27], C_44_Mab-3 (an anti-CD44v5 mAb) [28], C_44_Mab-9 (an anti-CD44v6 mAb) [29], C_44_Mab-34 (an anti-CD44v7/8 mAb) [30], and C_44_Mab-1 (an anti-CD44v9 mAb) [31]. The generation of anti-CD44 mAbs, which recognize all variant exons, is important for the comprehensive analysis of human tumors.

In this study, we established a novel anti-CD44v10 mAb, C_44_Mab-18 (IgM, kappa), using the CBIS method, and evaluated its applications via flow cytometry, Western blotting, and immunohistochemical analyses of OSCC tissues.

## 2. Materials and Methods

### 2.1. Cell Lines

A human pancreatic cancer cell line (PANC-1, the Cell Resource Center for Biomedical Research Institute of Development, Aging Sendai, Japan), a mouse multiple myeloma P3x63Ag8U.1 (P3U1) and Chinese hamster ovary (CHO)-K1 cell lines (the American Type Culture Collection, Manassas, VA, USA) were cultured using RPMI-1640 medium (Nacalai Tesque, Inc., Kyoto, Japan) containing 10% heat-inactivated fetal bovine serum (FBS; Thermo Fisher Scientific, Inc., Waltham, MA, USA) and antibiotics (100 U/mL penicillin, 100 μg/mL streptomycin, and 0.25 μg/mL amphotericin B). A human OSCC cell line (HSC-3, the Japanese Collection of Research Bioresources, Osaka, Japan) was cultured in Dulbecco’s Modified Eagle Medium (DMEM) (Nacalai Tesque, Inc., Kyoto, Japan) supplemented as indicated above. All cell lines were grown in a humidified incubator at 37 °C with 5% CO_2_.

The cDNAs of CD44v3–10 and CD44s were obtained as described previously [20]. The cDNAs were cloned into pCAG-zeo-ssPA16 and pCAG-neo-ssPA16 vectors with a signal sequence and N-terminal PA16 tag (GLEGGVAMPGAEDDVV). The PA16 tag can be detected by NZ-1 mAb, which was originally developed as an anti-human podoplanin (PDPN) mAb [32]. Stable transfectants including PANC-1/CD44v3–10, CHO/CD44v3–10, and CHO/CD44s were established by introducing corresponding vectors into the cells using a Neon transfection system (Thermo Fisher Scientific, Inc.).

### 2.2. Production of Hybridoma Cells

PANC-1/CD44v3–10 (1 × 10^8^ cells) was intraperitoneally administrated into the 6-week-old female BALB/c mice (CLEA Japan, Tokyo, Japan) with Imject Alum (Thermo Fisher Scientific Inc.). Additional three times immunizations of PANC-1/CD44v3–10 (1 × 10^8^ cells) and a booster injection of PANC-1/CD44v3–10 (1 × 10^8^ cells) two days before the sacrifice was performed. Hybridomas were produced as described previously [28]. The supernatants were selected by flow cytometer (SA3800 Cell Analyzer) and SA3800 software (ver. 2.05, Sony Corp. Tokyo, Japan).

### 2.3. Enzyme–Linked Immunosorbent Assay (ELISA)

Thirty-four peptides, which cover the variant region of CD44v3–10 [22], were obtained from Sigma-Aldrich Corp. (St. Louis, MO, USA), and immobilized on Nunc Maxisorp immunoplates (Thermo Fisher Scientific Inc.) at 20 µg/mL. After the blocking with 1% (*w*/*v*) bovine serum albumin (BSA) in phosphate-buffered saline (PBS) containing 0.05% (*v*/*v*) Tween 20 (PBST; Nacalai Tesque, Inc.), C_44_Mab-18 (1 µg/mL) was added to each well. The wells were further treated with anti-mouse immunoglobulins peroxidase-conjugate (1:2000 diluted; Agilent Technologies Inc., Santa Clara, CA, USA). The enzymatic reaction was performed using an ELISA POD Substrate TMB Kit (Nacalai Tesque, Inc.) The optical density (655 nm) was measured using an iMark microplate reader (Bio-Rad Laboratories, Inc., Berkeley, CA, USA).

### 2.4. Flow Cytometry

CHO/CD44v3–10, CHO-K1, and HSC-3 cells (1 × 10^5^ cells/sample) were incubated with C_44_Mab-18, C_44_Mab-46, or blocking buffer (0.1% BSA in PBS; control) for 30 min at 4 °C. The cells were further treated with anti-mouse IgG conjugated with Alexa Fluor 488 (1:2000; Cell Signaling Technology, Inc. Danvers, MA, USA), and analyzed as indicated above.

### 2.5. Determination of Apparent Dissociation Constant (K_D_) via Flow Cytometry

The serially diluted C_44_Mab-18 at the indicated concentrations was suspended with 2 × 10^5^ of HSC-3 and CHO/CD44v3–10 cells. The cells were further treated with anti-mouse IgG conjugated with Alexa Fluor 488 (1:200). Fluorescence data were analyzed, and the apparent dissociation constant (*K*_D_) was determined by fitting binding isotherms to built-in one-site binding models of GraphPad Prism 8 (GraphPad Software, Inc., La Jolla, CA, USA).

### 2.6. Western Blot Analysis

The SDS-polyacrylamide gel for electrophoresis and transfer onto polyvinylidene difluoride membranes was achieved as described previously [28]. After the blocking in PBST containing 4% skim milk (Nacalai Tesque, Inc.), the membranes were incubated with 10 μg/mL of C_44_Mab-46, 10 μg/mL of C_44_Mab-18, or 0.5 μg/mL of an anti-β-actin mAb (AC-15; Sigma-Aldrich Corp.). The membranes were further treated with peroxidase-conjugated anti-mouse immunoglobulins (diluted 1:1000; Agilent Technologies, Inc.). Finally, the chemiluminescence signal was obtained using ImmunoStar LD (FUJIFILM Wako Pure Chemical Corporation, Osaka, Japan) and was detected using a Sayaca-Imager (DRC Co., Ltd., Tokyo, Japan).

### 2.7. Immunohistochemical Analysis of Formalin-Fixed Paraffin-Embedded (FFPE) Tissues

Antigen retrieval of an OSCC tissue array (OR601c; US Biomax Inc., Rockville, MD, USA) was performed using EnVision FLEX Target Retrieval Solution High pH (Agilent Technologies, Inc.). SuperBlock T20 (Thermo Fisher Scientific, Inc.) was used for blocking. The sections were incubated with 1 μg/mL of C_44_Mab-18 and 1 μg/mL of C_44_Mab-46 at room temperature for 1 h. The sections were further treated with the EnVision+ Kit for a mouse (Agilent Technologies Inc.) at room temperature for 30 min. The chromogenic reaction and counterstaining were performed using 3,3′-diaminobenzidine tetrahydrochloride (DAB; Agilent Technologies Inc.) and hematoxylin (FUJIFILM Wako Pure Chemical Corporation), respectively.

## 3. Results

### 3.1. Establishment of an Anti-CD44 mAbs via Immunization of PANC-1/CD44v3–10 Cells

**Figure 1:**
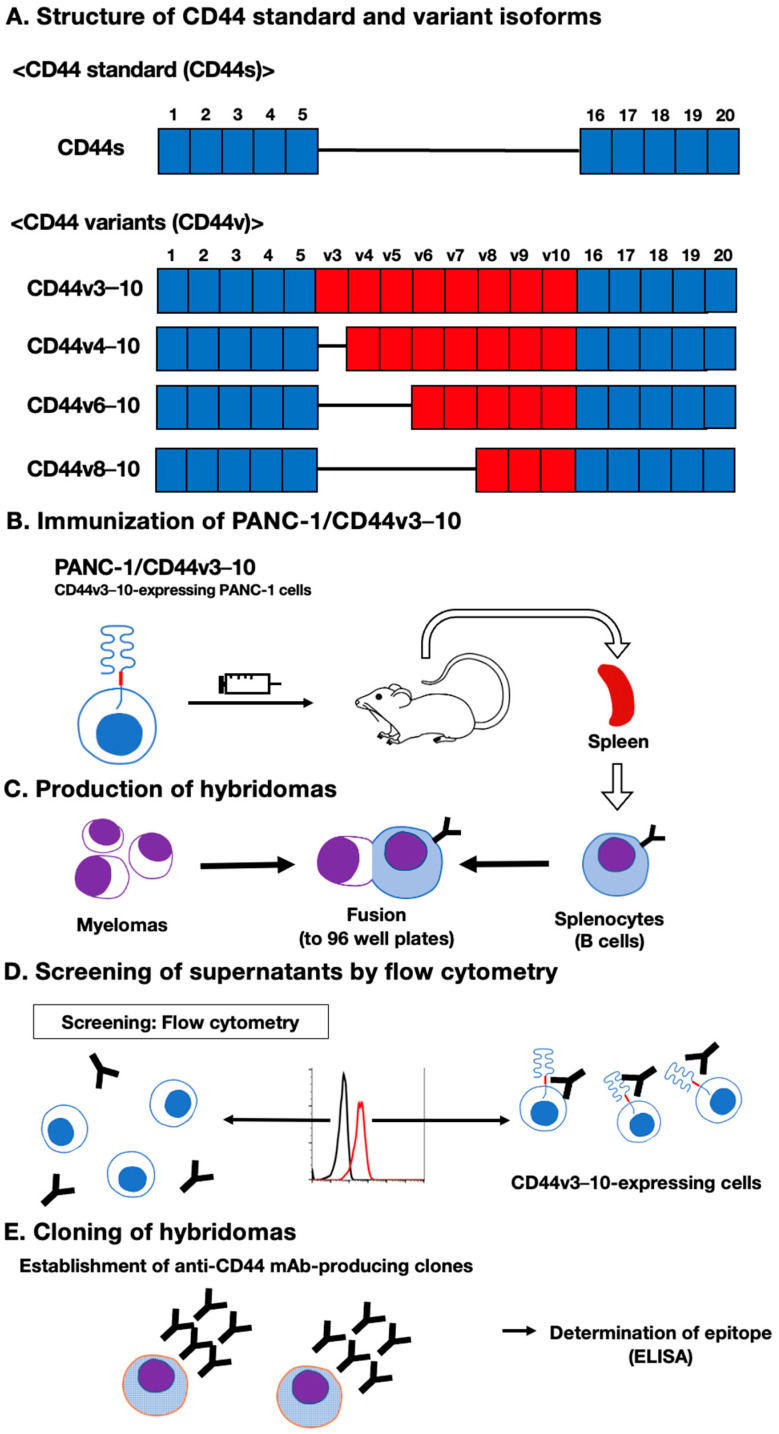
A schematic illustration of the CBIS method to establish anti-human CD44 mAbs. (**A**) Structure of CD44. The CD44s mRNA contains the constant exons (1 to 5) and (16 to 20). The CD44v including CD44v3–10, CD44v4–10, CD44v6–10, and CD44v8–10 are produced via the alternative splicing of variant exons. (**B**) PANC-1/CD44v3–10 cells were injected into BALB/c mice intraperitoneally. (**C**) Hybridomas were produced via the fusion of the splenocytes and P3U1 cells (**D**) The screening was performed via flow cytometry using CHO/CD44v3–10 and parental CHO-K1 cells. (**E**) A clone C_44_Mab-18 (IgM, kappa) was established after cloning.

In our previous work, we have established anti-CD44 mAbs, including C_44_Mab-5 (pan-CD44) [20], C_44_Mab-6 (v3) [26], C_44_Mab-3 (v5) [28], C_44_Mab-9 (v6) [29], and C_44_Mab-1 (v9) [31], using CHO/CD44v3–10 cells as an immunogen. In this study, we established another stable transfectant (PANC-1/CD44v3–10 cells) (Figure 1A). Mice were immunized with PANC-1/CD44v3–10 cells (Figure 1B), and hybridomas were produced via fusion between the splenocyte and P3U1 cells (Figure 1C). The supernatants, which were reactive to CHO/CD44v3–10 cells, but not to CHO-K1, were selected via flow cytometry-based high throughput screening (Figure 1D). After cloning by limiting dilution, anti-CD44 mAb-producing clones were finally established (Figure 1E).

### 3.2. Flow Cytometric Analysis of C_44_Mab-18- to CD44-Expressing Cells

**Figure 2:**
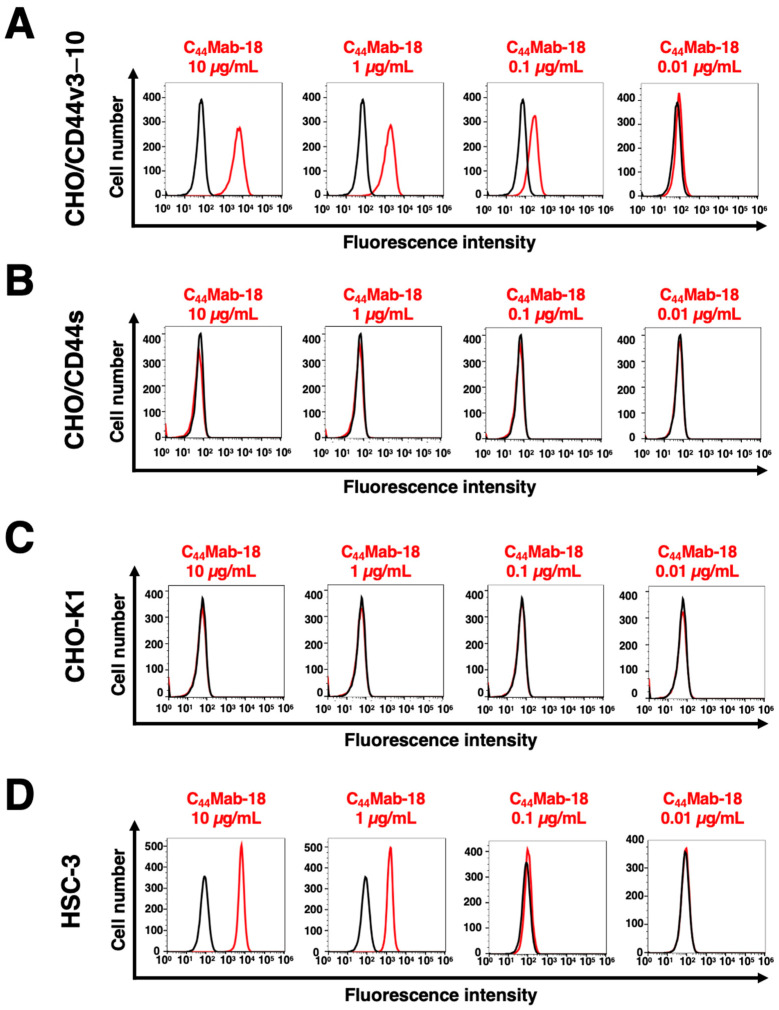
Flow cytometry using C_44_Mab-18. CHO/CD44v3–10 (**A**), CHO/CD44s (**B**), CHO-K1 (**C**), and HSC-3 (**D**) cells were treated with 0.01–10 µg/mL of C_44_Mab-18. Then, cells were treated with Alexa Fluor 488-conjugated anti-mouse IgG (Red line). The black line represents the negative control (blocking buffer).

In this study, established clones, the epitope of which includes CD44v10, were mainly determined to be IgM, although all mAbs against other CD44 variants are IgG [26,27,28,29,30,31]. Among those clones, we examined the reactivity of C_44_Mab-18 (IgM, kappa) against CHO/CD44v3–10 and CHO/CD44s cells via flow cytometry. C_44_Mab-18 dose-dependently recognized CHO/CD44v3–10 cells (Figure 2A). In contrast, C_44_Mab-18 recognized neither CHO/CD44s (Figure 2B) nor CHO-K1 (Figure 2C) cells. We confirmed that an anti-pan-CD44 mAb, C_44_Mab-46 [21], recognized CHO/CD44s cells, but not CHO-K1 cells (Supplementary Figure S1). Furthermore, C_44_Mab-18 could recognize HSC-3 cells (Figure 2D) in a dose-dependent manner. These results indicated that C_44_Mab-18 recognizes the variant exon-encoded region between v3 and v10 (Figure 1A).

### 3.3. Epitope Mapping of C_44_Mab-18 by ELISA

**Figure 3:**
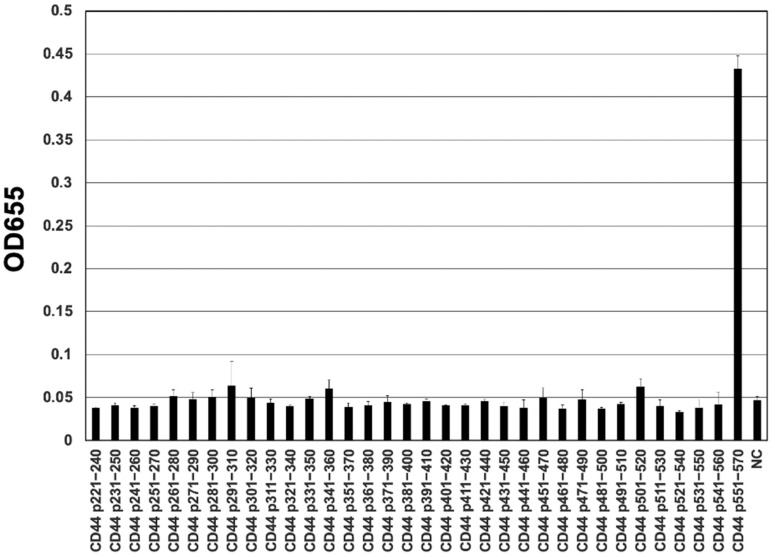
Determination of C_44_Mab-18 epitope using ELISA. The synthesized peptides, that cover the variant exon-encoded region between v3 and v10, were immobilized on immunoplates. The plates were incubated with C_44_Mab-18, followed by incubation with anti-mouse immunoglobulins -conjugated with peroxidase. Optical density (655 nm) was measured. The CD44 p551–570 sequence (SNSNVNRSLSGDQDTFHPSG) corresponds to variant 10 and the constant exon 16-encoded sequence. Error bars represent means ± SDs. NC, negative control (0.1% DMSO [solvent] in PBS).

To determine the epitope of C_44_Mab-18, we performed the ELISA using synthetic peptides, which cover the variant exon-encoded region between v3 and v10 [22]. As shown in Figure 3, C_44_Mab-18 recognized the CD44 p551–570 peptide (SNSNVNRSLSGDQDTFHPSG), which corresponds to variant 10 and constant exon 16-encoded sequence (Supplementary Table S1). In contrast, C_44_Mab-18 never recognized other v3- and v10-encoded peptides. This and the results in Figure 2 indicate that C_44_Mab-18 specifically recognizes the variant 10-containing CD44.

### 3.4. Determination of the Apparent Binding Affinity of C_44_Mab-18 via Flow Cytometry

**Figure 4:**
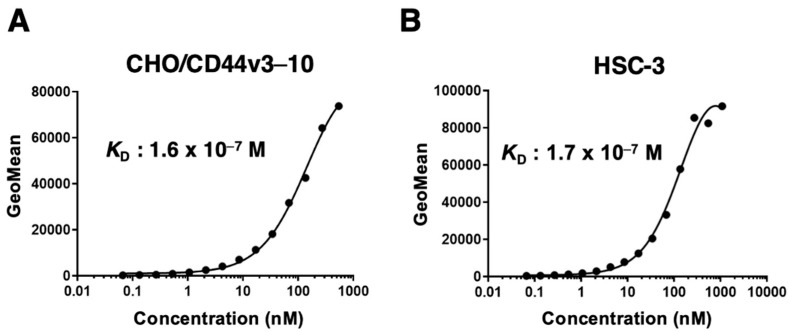
The determination of the apparent dissociation constant (*K*_D_) of C_44_Mab-18. C_44_Mab-18 was treated with CHO/CD44v3–10 at indicated concentrations (**A**) and with HSC-3 (**B**). The cells were treated with anti-mouse IgG conjugated with Alexa Fluor 488. Fluorescence data were collected, followed by the calculation of *K*_D_ using GraphPad PRISM 8.

We measured the apparent binding affinity of C_44_Mab-18 to CHO/CD44v3–10 and HSC-3 cells. The apparent dissociation constant (*K*_D_) of C_44_Mab-18 for CHO/CD44v3–10 (Figure 4A) and HSC-3 (Figure 4B) was 1.6 × 10^−7^ M and 1.7 × 10^−7^ M, respectively. These results indicated that C_44_Mab-18 possesses a moderate binding affinity for CD44v3–10 or endogenous CD44v10-expressing cells.

### 3.5. Western Blot Analysis

**Figure 5:**
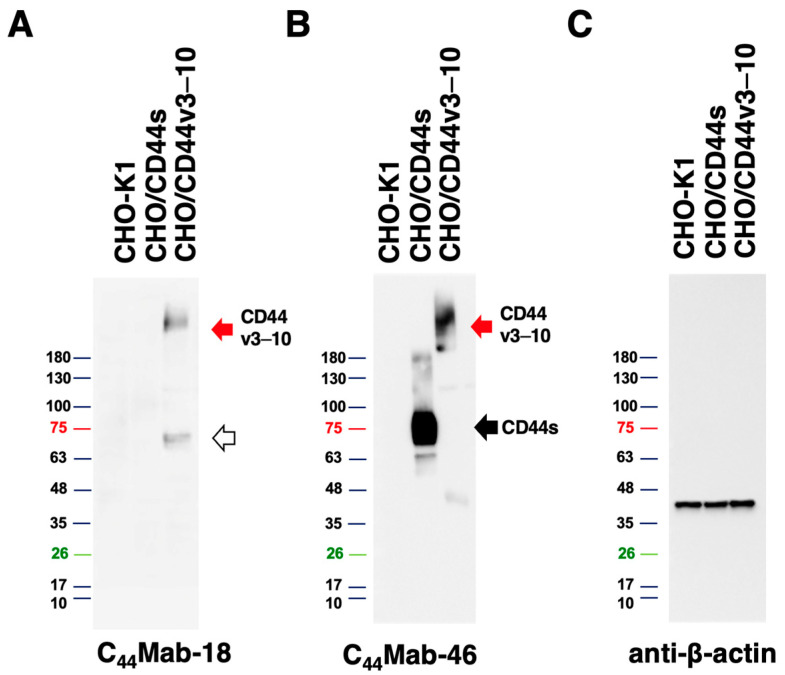
Western blot analysis using C_44_Mab-18. The total cell lysates (10 µg of protein) were separated and transferred onto polyvinylidene difluoride (PVDF) membranes. The membranes were incubated with 10 µg/mL of C_44_Mab-18 (**A**), 10 µg/mL of C_44_Mab-46 (**B**), or 0.5 µg/mL of an anti-β-actin mAb (**C**), followed by incubation with peroxidase-conjugated anti-mouse immunoglobulins. The red arrows indicate the CD44v3–10 (>180 kDa). The black arrow indicates the CD44s (~75 kDa). The white arrow indicates a lower molecular weight band recognized by C_44_Mab-18 in CHO/CD44v3–10 lysate (~70 kDa).

To assess the sensitivity of C_44_Mab-18 in Western blot analysis, we analyzed the cell lysates from CHO-K1, CHO/CD44s, and CHO/CD44v3–10. C_44_Mab-18 mainly detected CD44v3–10 as more than 180-kDa and ~70-kDa bands. However, C_44_Mab-18 did not detect any bands from lysates of CHO-K1 and CHO/CD44s cells (Figure 5A). An anti-pan-CD44 mAb, C_44_Mab-46, recognized both CD44v3–10 (>180 kDa) and CD44s (~75 kDa) bands in the lysates of CHO/CD44v3–10 and CHO/CD44s, respectively (Figure 5B). We used β-actin as a loading control (Figure 5C). These results indicate that C_44_Mab-18 can detect exogenous CD44v3–10.

### 3.6. Immunohistochemical Analysis Using C_44_Mab-18 against Tumor Tissues

**Figure 6:**
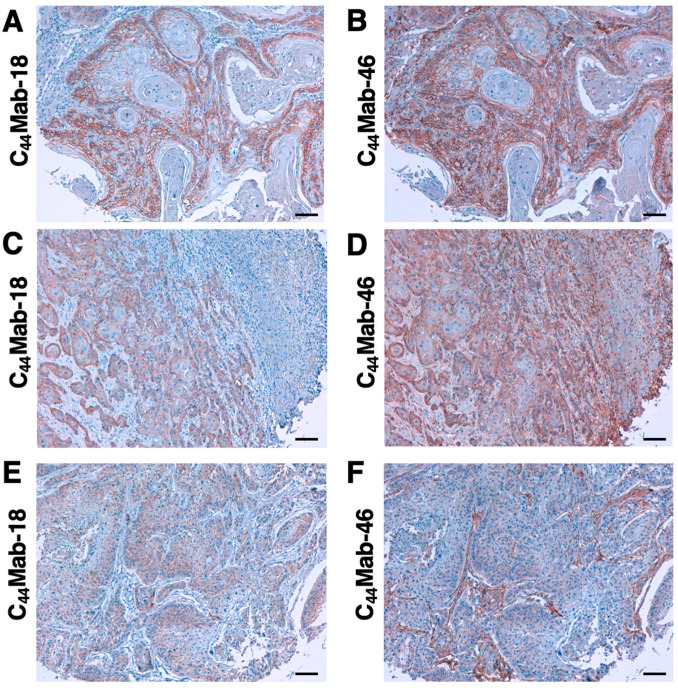
Immunohistochemical analysis using C_44_Mab-18 and C_44_Mab-46 against FFPE OSCC tissues. (**A**–**F**) Serial sections of the OSCC tissue array (OR601c) were incubated with 1 µg/mL of C_44_Mab-18 or C_44_Mab-46 followed by treatment with the Envision+ kit. The chromogenic reaction and counterstaining were performed using 3,3′-diaminobenzidine tetrahydrochloride and hematoxylin, respectively. Scale bar = 100 µm.

Since HNSCC is revealed as the second highest CD44-expressing tumor in the Pan-Cancer Atlas [5], we examined the reactivity of C_44_Mab-18 and C_44_Mab-46 in immunohistochemical analyses using FFPE sections of OSCC tissue array. As shown in Figure 6, C_44_Mab-18 was able to distinguish tumor cells from stromal tissues. In contrast, C_44_Mab-46 stained both. We summarized the data of immunohistochemical analyses in Table 1; C_44_Mab-18 stained 41 out of 50 cases (82%) in OSCC. These results indicate that C_44_Mab-18 applies to the immunohistochemical analysis of FFPE tumor sections.

## 4. Discussion

In our previous work, we established anti-CD44 mAbs using CHO/CD44v3–10 [20,26,28,29,31] and purified CD44v3–10 ectodomain [21,30] as immunogens. In this study, we used PANC-1/CD44v3–10 as another immunogen. We have compiled a list with this information in “Antibody Bank” (see Supplementary Materials). In this study, we listed a novel anti-CD44v antibody C_44_Mab-18, which recognizes the border sequence between variant 10 and constant exon 16 (Figure 3). Furthermore, C_44_Mab-18 could recognize CHO/CD44v3–10, but not CHO/CD44s in flow cytometry (Figure 2) and Western blot analyses (Figure 5). Moreover, C_44_Mab-18 could stain tumor cells, but not stromal tissues, which could be stained by C_44_Mab-46, an anti-pan-CD44 mAb (Figure 6). These results indicate that C_44_Mab-18 is an anti-CD44v10 mAb.

The VFF series anti-human CD44v mAbs were previously established via the immunization of glutathione *S*-transferase fused CD44v3–10 produced by bacteria [33,34]. The clones, VFF-8 (anti-CD44v5), VFF-18 (anti-CD44v6), VFF-9 (anti-CD44v7), VFF-17 (anti-CD44v7/8), and VFF-14 (anti-CD44v10) have been used for various applications [35]. Although VFF-14 was shown to apply to immunohistochemistry [36], the detailed binding epitope of VFF-14 has not been reported. In this study, we determined the epitope of C_44_Mab-18 as the CD44 p551–570 peptide (SNSNVNRSLSGDQDTFHPSG), which corresponds to the variant 10 (underlined) and constant exon 16-encoded region. In contrast, C_44_Mab-18 never recognizes the p541–560 peptide (FGVTAVTVGDSNSNVNRSLS) in the variant 10 region. Therefore, C_44_Mab-18 could have the epitope in the border region, but the inclusion of variant 10 is essential for the recognition.

Since the CD44 protein is modified by a variety of *N*-glycans and *O*-glycans, the molecular weight of CD44v isoforms surpasses 200-kDa [37]. C_44_Mab-18 recognized both more than 180-kDa and ~70-kDa bands (Figure 5A) in the lysate from CHO/CD44v3–10. The 70 kDa is approximately identical to the predicted molecular weight of CD44v3–10 from the amino acid sequence. Therefore, C_44_Mab-18 could recognize CD44v3–10 regardless of the glycosylation. The detailed epitope mapping and the influence of glycosylation on C_44_Mab-18 recognition should be investigated in future studies.

CD44v8–10 was shown to interact with xCT, a glutamate–cystine transporter, and regulate the level of reduced glutathione in tumor cells. The interaction is important for the stabilization of xCT on the cell surface, which promotes the defense against reactive oxygen species [17]. Furthermore, the interaction failed in CD44v8–10 (S301A), an *N*-linked glycosylation consensus motif (Asn-X-Ser/Thr) mutant in the variant 10-encoded region [17]. Therefore, it is worthwhile to investigate whether C_44_Mab-18 interferes with the interaction between CD44v8–10 and xCT in future studies. Furthermore, several studies have revealed that CD44v9 is used as a predictive marker for recurrence [38] and a biomarker for patient selection and efficacy of xCT inhibitors, sulfasalazine in gastric cancer [39]. Further investigations are also required to clarify the clinical significance of CD44v10 expression using C_44_Mab-18.

The mAbs against CD44 have been considered a therapeutic option for solid tumors and leukemia [12]. However, anti-pan-CD44 mAbs can affect normal tissues such as the epithelium and hematopoiesis. In a preclinical study using a murine thymoma model, a comparative study between an anti-pan-CD44 mAb (IM-7) and an anti-murine CD44v10 mAb (K926) was conducted in CD44v10-transfected EL4 thymoma (EL4-v10) [40]. The results showed that a blockade of CD44v10 by K926 was superior to that of IM-7 in intra-marrow EL4-v10 growth retardation. Furthermore, K926 hardly disturbed the hematopoietic stem cell (HSC) interaction with the bone marrow stroma. In contrast, IM-7 strongly affected the embedding of HSC in the bone marrow stroma [40]. These results indicated that the therapeutic use of anti-pan-CD44 mAbs should be avoided in favor of CD44v-specific mAbs as far as leukemic cells express CD44v isoforms.

In a humanized mouse model, CD44v8–10 was elevated during chronic myeloid leukemia progression from chronic phase to blast crisis [41]. Furthermore, increased transcription of CD44 mRNA was observed in human acute myeloid leukemia (AML) patients with *FLT3* or *DNMT3A* mutations through the suppression of CpG islands methylation in the promoter [42]. An anti-CD44v6 mAb (BIWA-8) derived from VFF-18 [43] was engineered to develop chimeric antigen receptors (CARs) for AML with *FLT3* or *DNMT3A* mutations. The CD44v6 CAR-T cells exhibited potent anti-leukemic effects [42]. We have established class-switched and defucosylated IgG_2a_ recombinant mAbs and evaluated the antitumor activity in xenograft models [44]. Therefore, the production of class-switched and defucosylated C_44_Mab-18 is an important strategy to evaluate the antitumor effect in preclinical models.

Since anti-pan-CD44 and anti-CD44v mAbs still have the possibility of causing side effects by affecting normal tissues, the clinical applications are limited. This study used tumor cell-expressed CD44v3–10 as an immunogen. This strategy is critical for the development of cancer-specific mAbs (CasMabs). We developed podocalyxin-targeting CasMabs [45] and PDPN-targeting CasMabs [46], which react with the aberrantly glycosylated targets selectively expressed in cancer [47]. Anti-PDPN-CasMabs have been applied to CAR-T therapy in preclinical studies [48,49,50]. For CasMab development, we should perform a further selection of our established anti-CD44 mAbs by comparing the reactivity against normal cells and tissues. Anti-CD44 CasMabs could be applicable for designing the modalities, including antibody-drug conjugates and CAR-T.

## 5. Conclusions

In this study, we established an anti-CD44v10 mAb (C_44_Mab-18). We also established an anti-CD44v8 mAb (C_44_Mab-94) (manuscript submitted, see Supplementary Materials). Therefore, we have established an anti-CD44 mAb library that covers almost all CD44 variants. This library could contribute to the diagnosis of not only carcinoma, but also hematopoietic malignancies. Since we have already cloned the V_H_ and V_L_ cDNA of anti-CD44 mAbs, the production of recombinant mAbs or CARs could contribute to the development of novel tumor therapies.

## Figures and Tables

**Table 1:** Immunohistochemical analysis using C_44_Mab-18 against OSCC tissue array.

No.	Age	Sex	Organ /Anatomic Site	Pathology Diagnosis	TNM	C_44_Mab-18	C_44_Mab-46
1	78	M	Tongue	SCC of tongue	T2N0M0	+	+
2	40	M	Tongue	SCC of tongue	T2N0M0	+	++
3	75	F	Tongue	SCC of tongue	T2N0M0	-	+
4	35	F	Tongue	SCC of tongue	T2N0M0	++	++
5	61	M	Tongue	SCC of tongue	T2N0M0	++	+++
6	41	F	Tongue	SCC of tongue	T2N0M0	+	+
7	64	M	Tongue	SCC of right tongue	T2N2M0	++	++
8	76	M	Tongue	SCC of tongue	T1N0M0	++	++
9	50	F	Tongue	SCC of tongue	T2N0M0	++	++
10	44	M	Tongue	SCC of tongue	T2N1M0	++	+++
11	53	F	Tongue	SCC of tongue	T1N0M0	+	++
12	46	F	Tongue	SCC of tongue	T2N0M0	++	+
13	50	M	Tongue	SCC of root of tongue	T3N1M0	++	+
14	36	F	Tongue	SCC of tongue	T1N0M0	++	+++
15	63	F	Tongue	SCC of tongue	T1N0M0	+	+
16	46	M	Tongue	SCC of tongue	T2N0M0	+	-
17	58	M	Tongue	SCC of tongue	T2N0M0	+	+
18	64	M	Lip	SCC of lower lip	T1N0M0	+	+++
19	57	M	Lip	SCC of lower lip	T2N0M0	+	+++
20	61	M	Lip	SCC of lower lip	T1N0M0	+	++
21	60	M	Gum	SCC of gum	T3N0M0	++	+
22	60	M	Gum	SCC of gum	T1N0M0	+++	+++
23	69	M	Gum	SCC of upper gum	T3N0M0	++	++
24	53	M	Bucca cavioris	SCC of bucca cavioris	T2N0M0	++	+
25	55	M	Bucca cavioris	SCC of bucca cavioris	T1N0M0	+++	+
26	58	M	Tongue	SCC of base of tongue	T1N0M0	++	++
27	63	M	Oral cavity	SCC	T1N0M0	+++	++
28	48	F	Tongue	SCC of tongue	T1N0M0	+	+
29	80	M	Lip	SCC of lower lip	T1N0M0	+++	+++
30	77	M	Tongue	SCC of base of tongue	T2N0M0	++	++
31	59	M	Tongue	SCC of tongue	T2N0M0	+	-
32	77	F	Tongue	SCC of tongue	T1N0M0	+	++
33	56	M	Tongue	SCC of root of tongue	T2N1M0	+	+
34	60	M	Tongue	SCC of tongue	T2N1M0	++	++
35	62	M	Tongue	SCC of tongue	T2N0M0	+	++
36	67	F	Tongue	SCC of tongue	T2N0M0	-	++
37	47	F	Tongue	SCC of tongue	T2N0M0	+++	+++
38	37	M	Tongue	SCC of tongue	T2N1M0	-	-
39	55	F	Tongue	SCC of tongue	T2N0M0	+	+
40	56	F	Bucca cavioris	SCC of bucca cavioris	T2N0M0	+	+
41	49	M	Bucca cavioris	SCC of bucca cavioris	T1N0M0	-	-
42	45	M	Bucca cavioris	SCC of bucca cavioris	T2N0M0	-	-
43	42	M	Bucca cavioris	SCC of bucca cavioris	T3N0M0	+++	++
44	44	M	Jaw	SCC of right drop jaw	T1N0M0	+	+++
45	40	F	Tongue	SCC of base of tongue	T2N0M0	-	++
46	49	M	Bucca cavioris	SCC of bucca cavioris	T1N0M0	++	+++
47	56	F	Tongue	SCC of base of tongue	T2N0M0	-	+
48	42	M	Bucca cavioris	SCC a of bucca cavioris	T3N0M0	+++	+++
49	87	F	Face	SCC a of left face	T2N0M0	-	+
50	50	M	Gum	SCC of gum	T2N0M0	-	-

-, No stain; +, Weak intensity; ++, Moderate intensity; +++, Strong intensity.

## Data Availability

All related data and methods are presented in this paper. Additional inquiries should be addressed to the corresponding authors.

## Supplementary Materials

The following supporting information can be downloaded at: https://www.mdpi.com/article/10.3390/cimb45070333/s1. Supplementary Figure S1, Recognition of CHO/CD44s and CHO/CD44v3–10 by C_44_Mab-46 by flow cytometry. Supplementary Table S1, The determination of the binding epitope of C_44_Mab-18 by ELISA. The information on anti-CD44 mAbs in our laboratory is available in “Antibody Bank” [http://www.med-tohoku-antibody.com/topics/001_paper_antibody_PDIS.htm#CD44 (accessed on 14 June 2023)].
