# Supplementary material for: Establishment of a Novel Anti-CD44 Variant 10 Monoclonal Antibody C44Mab-18 for Immunohistochemical Analysis against Oral Squamous Cell Carcinomas
Source: Curr Issues Mol Biol. 2023 Jun 21;45(7):5248–62. doi: 10.3390/cimb45070333 (PMC10378409; doi:10.3390/cimb45070333)
Supplement: Supplementary file 1 [file cimb-45-00333-s001.zip › supplementary Table S1 C44Mab-18(v10).pdf]

Table S1. The determination of the binding epitope of C44Mab-18 by ELISA.

Peptide	Coding exon*	Sequence	C44Mab-18
CD44p221–240	5/v3	ATSTSSNTISAGWEPNEENE	–
CD44p231–250	v3	AGWEPNEENEDERDRHLSFS	–
CD44p241–260	v3	DERDRHLSFSGSGIDDDEDF	–
CD44p251–270	v3/v4	GSGIDDDEDFISSTISTTPR	–
CD44p261–280	v3/v4	ISSTISTTPRAFDHTKQNQD	–
CD44p271–290	v4	AFDHTKQNQDWTQWNPSHSN	–
CD44p281–300	v4	WTQWNPSHSNPEVLLQTTTR	–
CD44p291–310	v4/v5	PEVLLQTTTRMTDVDRNGTT	–
CD44p301–320	v4/v5	MTDVDRNGTTAYEGNWNPEA	–
CD44p311–330	v5	AYEGNWNPEAHPPLIHHEHH	–
CD44p321–340	v5	HPPLIHHEHHEEEETPHSTS	–
CD44p331–350	v5/v6	EEEETPHSTSTIQATPSSTT	–
CD44p341–360	v5/v6	TIQATPSSTTEETATQKEQW	–
CD44p351–370	v6	EETATQKEQWFGNRWHEGYR	–
CD44p361–380	v6	FGNRWHEGYRQTPREDSHST	–
CD44p371–390	v6/v7	QTPREDSHSTTGTAAASAHT	–
CD44p381–400	v6/v7	TGTAAASAHTSHPMQGRTTP	–
CD44p391–410	v7	SHPMQGRTTPSPEDSSWTDF	–
CD44p401–420	v7	SPEDSSWTDFFNPISHPMGR	–
CD44p411–430	v7/v8	FNPISHPMGRGHQAGRRMDM	–
CD44p421–440	v7/v8	GHQAGRRMDMDSSHSTTLQP	–
CD44p431–450	v8	DSSHSTTLQPTANPNTGLVE	–
CD44p441–460	v8	TANPNTGLVEDLDRTGPLSM	–
CD44p451–470	v8/v9	DLDRTGPLSMTTQQSNSQSF	–
CD44p461–480	v8/v9	TTQQSNSQSFSTSHEGLEED	–
CD44p471–490	v9	STSHEGLEEDKDHPTTSTLT	–
CD44p481–500	v9/v10	KDHPTTSTLTSSNRNDVTGG	–
CD44p491–510	v9/v10	SSNRNDVTGGRRDPNHSEGS	–
CD44p501–520	v10	RRDPNHSEGSTTLLEGYTSH	–
CD44p511–530	v10	TTLLEGYTSHYPHTKESRTF	–
CD44p521–540	v10	YPHTKESRTFIPVTSAKTGS	–
CD44p531–550	v10	IPVTSAKTGSFGVTAVTVGD	–

CD44p541–560	v10	FGVTAVTVGDSNSNVNRSLS	–
CD44p551–570	v10/16	SNSNVNRSLSGDQDTFHPSG	+

+, OD655≧0.4; －, OD655<0.1

*The CD44 exon-cording regions are illustrated in Figure 1.
