# Supplementary figures and images for: Establishment of a Novel Anti-CD44 Variant 10 Monoclonal Antibody C44Mab-18 for Immunohistochemical Analysis against Oral Squamous Cell Carcinomas
Source: Curr Issues Mol Biol. 2023 Jun 21;45(7):5248–62. doi: 10.3390/cimb45070333 (PMC10378409; doi:10.3390/cimb45070333)

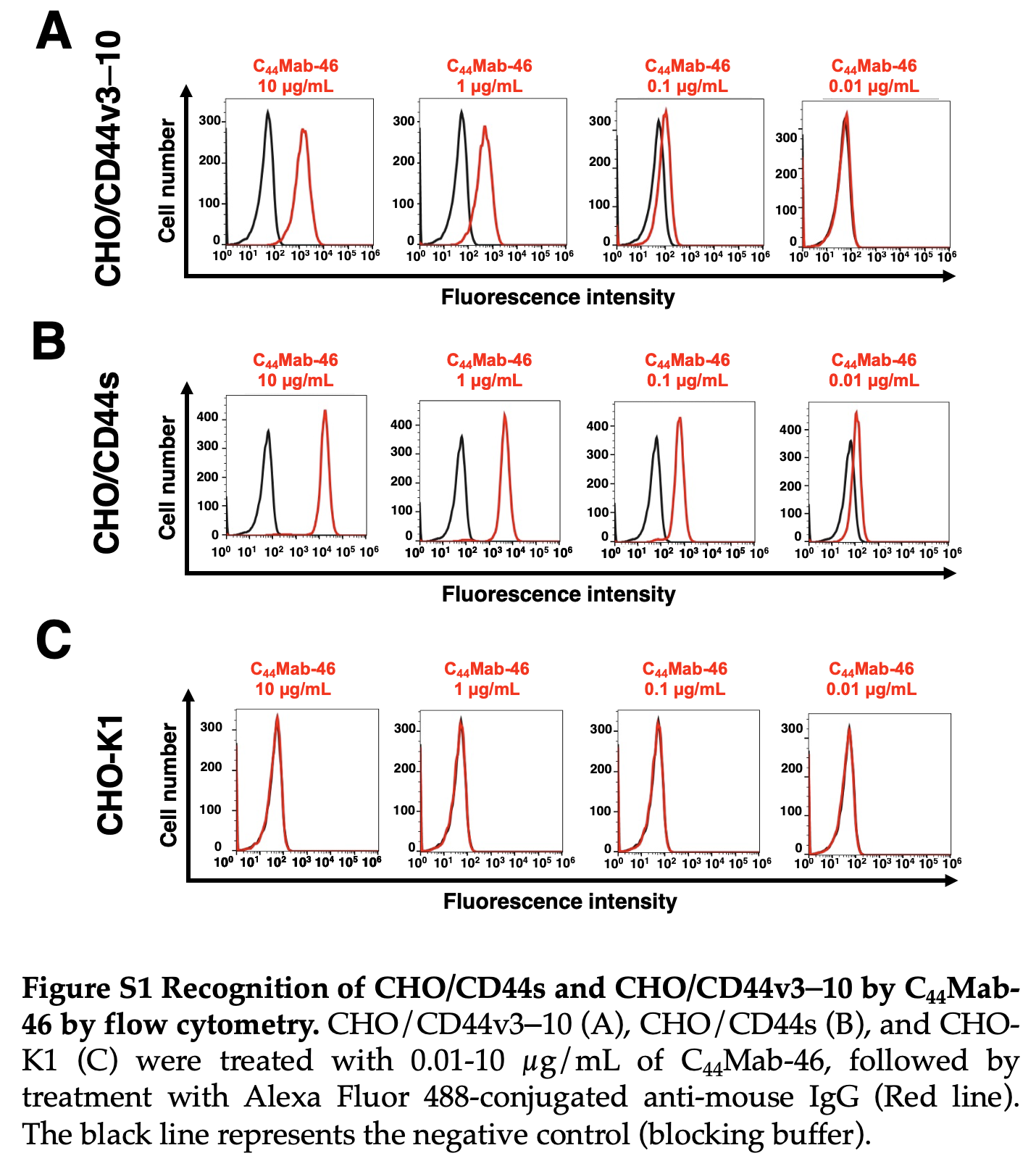

Supplement: Supplementary file 1 [file cimb-45-00333-s001.zip › supplementary Fig S1 C44Mab-18(v10).png]
